# Current progresses and challenges for microbiome research in human health: a perspective

**DOI:** 10.3389/fcimb.2024.1377012

**Published:** 2024-04-04

**Authors:** Simone Filardo, Marisa Di Pietro, Rosa Sessa

**Affiliations:** Department of Public Health and Infectious Diseases, Section of Microbiology, University of Rome “Sapienza”, Rome, Italy

**Keywords:** human microbiome, 16s metagenomic, whole genome sequencing, transcriptomics, metabolomics, proteomics, multi-omics

## Abstract

It is becoming increasingly clear that the human microbiota, also known as “the hidden organ”, possesses a pivotal role in numerous processes involved in maintaining the physiological functions of the host, such as nutrient extraction, biosynthesis of bioactive molecules, interplay with the immune, endocrine, and nervous systems, as well as resistance to the colonization of potential invading pathogens. In the last decade, the development of metagenomic approaches based on the sequencing of the bacterial 16s rRNA gene via Next Generation Sequencing, followed by whole genome sequencing via third generation sequencing technologies, has been one of the great advances in molecular biology, allowing a better profiling of the human microbiota composition and, hence, a deeper understanding of the importance of microbiota in the etiopathogenesis of different pathologies. In this scenario, it is of the utmost importance to comprehensively characterize the human microbiota in relation to disease pathogenesis, in order to develop novel potential treatment or preventive strategies by manipulating the microbiota. Therefore, this perspective will focus on the progress, challenges, and promises of the current and future technological approaches for microbiome profiling and analysis.

## 1 Introduction

The human organism hosts a wide variety of microorganisms, collectively known as the microbiota, capable to thrive in different bodily districts, including the gut, the skin, the lungs, the oral cavity, and the urogenital tract. Although most microorganisms (>90%) belonging to the resident microflora comes from the bacterial domain, also viruses, yeast and archaea coexist in the various sites of the human body, contributing to more than 150 times genetic information than that of the entire human genome (Grice and Segre, 2012; Hou et al., 2022).

For many years, bacterial culture has represented the primary approach for exploring the structure and function of the human microbiota, albeit with the important limitation that more than 99% of all microbes could not be cultured by standard laboratory techniques, especially a large number of anaerobic bacterial species (Rinke et al., 2013; Almeida et al., 2019). In 1996, the introduction of 16s ribosomal RNA gene polymerase chain reaction (PCR) has revolutionized the field of microbiota investigations; the first studies relied on molecular techniques such as the denaturing gradient gel electrophoresis (DGGE), the restriction fragment length polymorphism (RFLP), the terminal restriction fragment length polymorphism (TRFLP), and capillary sequencing of specific genes by Sanger’s methods (Eckburg et al., 2005; Thomas et al., 2012). These methodological approaches possessed severe challenges in addressing the complexity of the different microbial ecosystems, most importantly by showing only the presence of specific bacterial groups (Zoetendal et al., 2008; Carabeo-Pérez et al., 2019).

In this scenario, the development of metagenomic approaches based on the sequencing of the bacterial 16s rRNA gene via Next Generation Sequencing (NGS) has been, since around 2010, one of the great advances in molecular biology, allowing a better profiling of the human microbiota composition and, hence, a deeper understanding of the importance of microbiota in the etiopathogenesis of different pathologies (Carabeo-Pérez et al., 2019); indeed, in the past, most studies focused on the etiopathogenetic mechanisms underlying specific infectious agents, via *in vitro*, *ex vivo* or animal models, without acknowledging the important role of the microbiota (Di Pietro et al., 2015; Lenz and Dillard, 2018; Filardo et al., 2019b; Ansari and Yamaoka, 2022).

Thanks to advances in microbiota profiling through NGS, it is currently known that the human microbiota, also known as “the hidden organ”, possesses a pivotal role in numerous processes involved in maintaining the physiological functions of the host, such as nutrient extraction and biosynthesis of bioactive molecules, like vitamin, amino acids and lipids, interplay with the immune, endocrine, and nervous systems, namely the gut-organ axis, and resistance to the colonization of potential invading pathogens (Hanssen and Nieuwdorp, 2021; Hou et al., 2022; Saxami et al., 2023).

Given the key role of microbiota-host interplay in health and disease, it is becoming increasingly important to comprehensively characterize the composition and functional profiles of the human microbiota in relation to disease pathogenesis, in order to develop novel potential treatment or preventive strategies by manipulating the microbiota. Therefore, this perspective will focus on the progress, challenges, and promises of the current and future technological approaches for microbiome profiling and analysis.

## 2 Metagenomic sequencing of microbial communities

The most widely used technology for the profiling of human microbiota is the sequencing of hypervariable sub-regions of the 16s rRNA gene via NGS (Carabeo-Pérez et al., 2019). This is indeed a high-throughput sequencing technique that allows the simultaneous sequencing of a large number of short DNA fragments (150-250 base pairs) generated by amplification of a specific gene, or portion of a gene (Bonk et al., 2018). The hypervariable region, mainly the V4 or V3-V4, is considered for the characterization of bacterial taxa up to the genus level (Jones et al., 2022; Gao et al., 2023). NGS technologies include the pyrosequencing (454 Life Sciences), the massive sequencing via Illumina system, sequencing by ligation (SOLiD) and sequencing based on pH detection (Ion Torrent); amongst them, the Illumina sequencing platform has become the most extensively utilized in the field of microbiota research due to its high throughput and relatively low error rate, providing valuable contributions to the understanding of the complex interactions between the microbiota and the host (Heather and Chain, 2016; Carabeo-Pérez et al., 2019). However, the microbial profiling via metagenomic analysis of 16s rRNA gene has shown critical issues, including the unreliability of taxonomic classification at the species level, and the bias introduced by the sequencing platforms and the choice of diverse data processing software. Concerning the first aspect, different studies have highlighted that targeting one or few hypervariable sub-regions of the 16s rRNA gene may lead to the sub-optimal representation of certain bacterial species or even whole taxonomic groups, failing to capture the complex biodiversity of the human microbiota (López-Aladid et al., 2023). Moreover, the sequencing of short portions of the 16s rRNA gene hypervariable region, containing a total of 1542 base pairs, may miss relevant genetic variations outside of the sequenced area, leading to the impossibility to distinguish closely related bacterial species due to homology between sequences (Bailén et al., 2020). As for the second issue, it has been reported that the choice of different sequencing platform and data processing software might also introduce further variability, yielding differences in sample characterization depending on their accuracy, sensitivity, and error rate (Claesson et al., 2010, 2017). Similarly, the usage of different analytical pipelines applied to data processing might lead to differences in microbiota composition, as not all available pipelines include the same steps, and, to date, there is scarce literature on the advantages and disadvantages of available software packages (D’Argenio et al., 2014; Bailén et al., 2020).

In an attempt to overcome these limitations, full-length 16s rRNA gene sequencing, or whole-genome sequencing (WGS) via shotgun sequencing of all DNA fragments in a microbial community, have been developed (Johnson et al., 2019; Pérez-Cobas et al., 2020; Lin et al., 2021). In particular, the analysis of the full-length 16s rRNA gene sequence has provided evidence of additional variations at the species or strain levels, that is otherwise unseen through the regular short-read sequencing generally used in amplicon-based methods, leading to a more in-depth taxonomic classification of the human microbiota (Johnson et al., 2019; Bailén et al., 2020). For example, a higher resolution at the species level was obtained via full length 16s rRNA gene sequencing in patients with either *Mycoplasma pneumoniae* or unknown aetiology pneumonia, revealing high abundances of coinfections, or the presence of pathogenic bacteria like *Campylobacter concisus*, *Solobacterium moorei*, etc (Li et al., 2023b).

The WGS has, instead, enabled the identification of microbial functional genes by assembling all sequenced DNA fragments in a sample, making it possible to determine nearly the whole genetic potential of the previously uncharacterized microbial species (Pérez-Cobas et al., 2020). For example, WGS analysis has the capability to investigate, besides the composition of the microbiota, the presence of bacterial virulence factors as well as genes associated to drug-resistance; on this regard, a study has evidenced the higher abundances of adhesion, toxin, and type-III secretion system genes, alongside genes associated to tetracycline resistance, in the gut microbiota from HIV-1 positive patients taking antiretroviral therapy as compared to healthy controls (Bai et al., 2021). Furthermore, WGS has revealed the absence of drug-resistance genes in a novel marine multi-stress-tolerant aromatic yeast *Pichia kudriavzevii* HJ2, supporting its use as probiotic for its capability to achieve a 79.98% cholesterol removal rate, with a strong antioxidant capacity (Li et al., 2023c).

The full-length sequencing of 16s rRNA gene and the WGS has been rendered possible by the introduction of the so-called “third generation sequencing” technologies, such as in PacBio or Nanopore platforms (Carabeo-Pérez et al., 2019). Third generation sequencing provides the nucleotide sequence by reading at the single molecule level and avoiding, thus, the DNA amplification steps required for NGS technologies. By doing this, these methodological approaches have the capability to produce much longer reads than NGS methods, and to reduce the bias inherent in the PCR amplification, known to under-represent sequences from less abundant bacteria (van Dijk et al., 2018).

Long-read sequencing approaches hold the potential for improving metagenomic assembly, covering repetitive and low-coverage regions, and, hence, increasing assembly contiguity. This has facilitated the development of high-quality genome assemblies for the majority of microbes populating the human microbiota, although assembling all genomes of all bacteria within a single microbial niche remains challenging, due to the relatively shallow sequencing depth generally used in most studies (5-10 Giga base pairs, Gbp). To address this issue, the combination of long-read with short-read sequencing approaches might be a valid alternative for increasing coverage of low-abundance species, and consequently boosting assembly performance. This might enable a much more comprehensive overview of microbiota composition and, hence, a deeper understanding of the complex interactions between the different microbial communities and the host, although with the biggest disadvantage of a much higher (15-20 times more) cost than short-read sequencing approaches (Jin et al., 2022; Yue et al., 2023).

The main characteristics of long-read sequencing approaches lie in the production of a massive volume of complex data, with high throughput and multiple redundancies, making it challenging to efficiently analyse via traditional computational approaches and algorithms (Lin et al., 2021). Therefore, new bioinformatic tools are being developed, and, amongst them, machine learning (ML) has recently acquired great importance (Lin et al., 2021). ML are deep-learning algorithms able to learn from experience and discovery of data, exploring potential patterns and relationships that are difficult to infer via traditional methods (Li et al., 2022). Despite their potentials, ML models are computationally intensive, requiring more efficient computing facilities that are not often available to researchers, and are challenging to use as the primary method for analyzing metagenomic data due to the need for highly trained bioinformaticians (Frank et al., 2020); however, ML models are increasingly used in cutting-edge research and represent the path forward for investigating the growing complexity of the intricate interaction between microbiota and disease pathogenesis (Lin et al., 2021).

## 3 Towards a multi-omics approach

Current approaches for studying the microbiota are generally limited to only one or few aspects, typically the microbiota profiling through 16s rRNA amplicon sequencing, or through WGS via shotgun metagenomic. By contrast, the role of the human microbiota in disease pathogenesis involves a complex multi-dimensional network characterized by structural (the profiling of microbial composition) and functional (the determination of whole genetic pathways) interactions with the host, as well as by various small molecules, including metabolites, catabolites, and signal molecules, through which they interact (Chetty and Blekhman, 2024).

Indeed, it is becoming increasingly clear that the full scope of the multi-layered interplay between microorganisms and the host, affecting health outcomes, can only be revealed by integrating a multitude of different data layers along with the metagenomic analysis of the microbiome, leading to a multi-omics approach (Chetty and Blekhman, 2024). Multi-omics is defined as the integration of metagenomics with meta-transcriptomics, meta-proteomics, and metabolomics. Meta-transcriptomics can be performed via short- or long- read sequencing of mRNA, usually after filtering out highly abundant ribosomal rRNA, whereas both meta-proteomics and metabolomics data are generated via mass spectrometry. Specifically, meta-transcriptomics can provide insights into the microbial genetic pathways, by quantifying the abundance of transcripts in a sample reflecting bacterial gene expression activity (Aitmanaitė et al., 2023); Meta-proteomics is complementary to metagenomics and meta-transcriptomic since it reflects the activity of cellular translational and post-translational processes, by measuring the levels of all the proteins synthesized by the microbiota and the host, and, hence, providing insights into the functional role of microorganisms in host health (Hardouin et al., 2021); Metabolomics allow researchers to acquire a better understanding of metabolic and biochemical processes involving the microbiota and the host, by determining the levels of all small molecules in the microenvironment (Bartsch et al., 2023). An interesting application of multi-omics is described in a study on the dopamine metabolism in the marine yeast *Meyerozyma guilliermondii* GXDK6, by using WGS for identifying the key genes for dopamine biosynthesis, and transcriptomics as well as proteomics for showing the level of activation of the related metabolic pathway (**Sun et al., 2024** **).**

Multi-omics possess their own unique set of challenges to face, including the variability of results due to different software pipelines, and the excessive reliance on a paucity of curated and publicly available microbiome databases, limiting the information only to well-characterized microorganisms, transcripts, proteins, and metabolites (Chetty and Blekhman, 2024). However, these approaches provide more comprehensive information than the study of a single aspect, since all these datasets can generate a 360-degree view of the complex interactome between the microbiota and the host, elucidating the relationship between different cellular processes, such as, for example, between mRNA and proteins, or between different type of biomolecules synthesized by the microbiota or the host.

## 4 Discussion

Thanks to advanced metagenomic analysis, the composition of human microbiota has been shown as highly variable across body sites, with the gut microbiota receiving considerable interest, followed by the microbiota in other districts, like the oral cavity and the cervicovaginal microenvironment (Hou et al., 2022).

In fact, in the last decade, the gut microbiota has been regarded as the most significant microbial community in maintaining individual health; it is more complex than other microbiota niches, for instance, the cervicovaginal microbiota, and accounts for approximately 10^13^ bacteria; the most dominant bacterial phyla are *Firmicutes* (60–80%) and *Bacteroidetes* (20–40%), while Proteobacteria, Actinobacteria, and Fusobacteria are in the minority (Filardo et al., 2022a). The balance between obligate and facultative anaerobes is essential for the integrity and function of the intestinal epithelial barrier; certain conditions, like antibiotic treatment or unhealthy lifestyles, may alter the gut microbiota composition, named dysbiosis, leading to the onset and development of different pathologies (Naito, 2024). For example, the overgrowth of potentially harmful *Proteobacteria*, including *Enterobacteriaceae*, has been linked to the emergence of a pro-inflammatory microenvironment contributing to the pathogenesis of Inflammatory Bowel Diseases, as well as cancer and metabolic disorders like obesity, dyslipidaemia, type-2 diabetes, and non-alcoholic fatty liver disease (Jia et al., 2023; Li et al., 2023a). Specifically, it has been suggested that the relationship between the two dominant phyla, expressed as Firmicutes/Bacteroidetes ratio, may be associate to the development of metabolic diseases induced by high-fat diet (Jia et al., 2023); also, certain bacteria in the intestine, such as *Escherichia coli*, *Campylobacter jejuni*, *Fusobacterium nucleatum*, and *Bacteroides fragilis*, have been described to express toxin proteins causing intracellular reactive oxygen species accumulation and DNA damage, and contributing to colorectal carcinogenesis (Li et al., 2023a). Furthermore, changes in pH values as well as the presence of histologic alterations of the gastric mucosa, as in chronic atrophic gastritis, were also associated to increased gut dysbiosis characterized by the prevalence of oral bacteria, like *Rothia mucilaginosa*, *Streptococcus salivarius* and *Granulicatella adiacens* (Filardo et al., 2022b).

Beside the gut microbiota, a growing body of evidence has also been provided for the microbial community populating the oral cavity, that can be divided into multiple habitats, including saliva, tongue, subgingival/supragingival plaque, etc., harboring substantially similar microbiota populations with small scale differences due to pH, contact with hard or soft tissue surfaces, and bacterial interactions (Segata et al., 2012). The major bacteria present in the oral microbiota belong to the phyla *Firmicutes*, *Proteobacteria*, and *Bacteroidetes*; in dysbiosis conditions, the microbial imbalance characterized by the predominance of *Porphyromonas gingivalis* has been associated with periodontal diseases, including periodontitis, a chronic inflammatory condition leading to tissue destruction (Demkovych et al., 2023).

Less explored, but equally relevant for its health promoting activities, is the cervicovaginal microbiota, that harbors the simplest microbial community in the human organism; in fact, this is characterized, in healthy reproductive-age women, by the predominance of one or a few bacterial species, mainly *Lactobacillus* spp. (over 80%), alongside other bacteria present in much lower abundances, including *Staphylococcus* spp., *Streptococcus* spp., and *Bifidobacterium* spp. (France et al., 2022). The depletion of *Lactobacillus* spp. and the overgrowth of anaerobic bacteria belonging to the genera *Gardnerella* spp., *Prevotella* spp., *Atopobium* spp., etc., is well known to increase the risk for acquiring infections by sexually transmitted pathogens like *Chlamydia trachomatis* and Human Papilloma Virus (Filardo et al., 2019a; Kaliterna et al., 2023).

Overall, all this evidence led interesting application for the development of novel therapeutic or prevention strategies, by manipulating the microbiome with interventions like the supplementation with pre- or pro-biotics, or the fecal transplant.

However, our knowledge of the complex network of interactions between resident microorganisms and the host is still limited, due to the numerous challenges that current microbiota studies have to face. In particular, most research, due to budget restraints, are limited to a partial analysis of human microbiota from a compositional point of view, lacking information on the intricate web of gene pathways, proteins and metabolic processes that may be essential for the discovery of its etiopathogenetic role.

**Figure 1:**
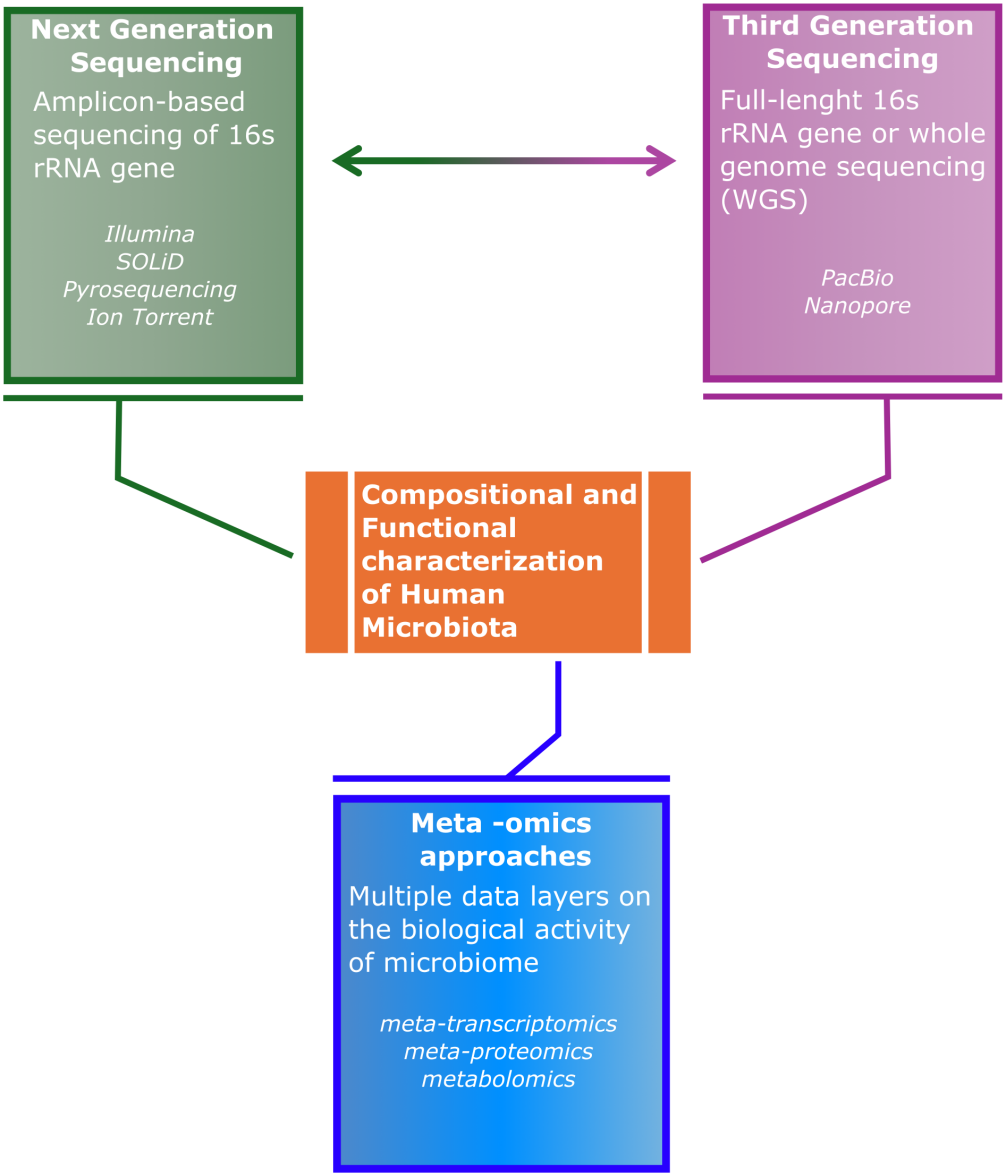
Schematic representation of the need for a better integration amongst Next Generation Sequencing, Third Generation Sequencing and -omics approaches, in order to reach a comprehensive characterization of the composition and functional aspect of microbiota-host interaction.

In the future, it will be essential to integrate multiple layers of information, from the analysis of gene transcripts to their protein products, as well as all the small molecules produced by microbial or host metabolism, in a process called multi-omics, with the aim to clearly describe the etiopathogenetic relationships between resident microorganisms and host disease conditions (**Figure 1**). On this regard, there are still important challenges to overcome: the need of increased availability of more advanced and comprehensive bioinformatic tools, allowing also non-bioinformaticians to extract useful information from meta-omics data, as well as a reduction in the analytical cost per sample so to increase its accessibility. Furthermore, another source of bias might be introduced by the absent standardization of sample collection from different sites, transportation, and storage, as well as by the heterogeneous methods for genomic DNA extraction, as they may introduce further variability in the quality of bacterial DNA isolation, alongside the partial removal of contaminating host DNA. Overall, improving on all these aspects will be fundamental steps to be taken for a future deep understanding of the intricate interplay between microbiome-host, potentially leading to the early diagnosis of chronic diseases for improved preventive and treatment approaches.

## Data availability statement

The original contributions presented in the study are included in the article/supplementary material. Further inquiries can be directed to the corresponding author.

## Author contributions

SF: Writing – review & editing, Writing – original draft, Conceptualization. MD: Writing – review & editing, Writing – original draft, Conceptualization. RS: Writing – review & editing, Writing – original draft, Supervision, Conceptualization.
